# Cell type discovery and representation in the era of high-content single cell phenotyping

**DOI:** 10.1186/s12859-017-1977-1

**Published:** 2017-12-21

**Authors:** Trygve Bakken, Lindsay Cowell, Brian D. Aevermann, Mark Novotny, Rebecca Hodge, Jeremy A. Miller, Alexandra Lee, Ivan Chang, Jamison McCorrison, Bali Pulendran, Yu Qian, Nicholas J. Schork, Roger S. Lasken, Ed S. Lein, Richard H. Scheuermann

**Affiliations:** 1 Allen Institute for Brain Science, Seattle, Washington, 98103 USA; 2 Department of Clinical Sciences, University of Texas Southwestern Medical Center, 5323 Harry Hines Blvd, Dallas, TX USA; 3 J. Craig Venter Institute, 4120 Capricorn Lane, La Jolla, CA 92037 USA; 4 Department of Pathology and Laboratory Medicine, Emory University, 201 Dowman Dr, Atlanta, GA 30322 USA; 5 Department of Pathology, University of California San Diego, 9500 Gilman Drive, La Jolla, CA 92093 USA

**Keywords:** Cell ontology, Single cell transcriptomics, Cell phenotype, Peripheral blood mononuclear cells, Neuron, Next generation sequencing, Cytometry, Open biomedical ontologies, Marker genes

## Abstract

**Background:**

A fundamental characteristic of multicellular organisms is the specialization of functional cell types through the process of differentiation. These specialized cell types not only characterize the normal functioning of different organs and tissues, they can also be used as cellular biomarkers of a variety of different disease states and therapeutic/vaccine responses. In order to serve as a reference for cell type representation, the Cell Ontology has been developed to provide a standard nomenclature of defined cell types for comparative analysis and biomarker discovery. Historically, these cell types have been defined based on unique cellular shapes and structures, anatomic locations, and marker protein expression. However, we are now experiencing a revolution in cellular characterization resulting from the application of new high-throughput, high-content cytometry and sequencing technologies. The resulting explosion in the number of distinct cell types being identified is challenging the current paradigm for cell type definition in the Cell Ontology.

**Results:**

In this paper, we provide examples of state-of-the-art cellular biomarker characterization using high-content cytometry and single cell RNA sequencing, and present strategies for standardized cell type representations based on the data outputs from these cutting-edge technologies, including “context annotations” in the form of standardized experiment metadata about the specimen source analyzed and marker genes that serve as the most useful features in machine learning-based cell type classification models. We also propose a statistical strategy for comparing new experiment data to these standardized cell type representations.

**Conclusion:**

The advent of high-throughput/high-content single cell technologies is leading to an explosion in the number of distinct cell types being identified. It will be critical for the bioinformatics community to develop and adopt data standard conventions that will be compatible with these new technologies and support the data representation needs of the research community. The proposals enumerated here will serve as a useful starting point to address these challenges.

## Background

Cells in multicellular organisms acquire specialized functions through the process of differentiation. This process is characterized by changes in gene expression through the actions of sequence-specific transcription factors and chromatin remodeling that results in a cell type-specific collection of messenger RNA transcripts expressed from a subset of genes in the organism’s genome. This transcriptional profile is then translated into a cell type-specific collection of proteins that corresponds to the functional parts list of the specialized cell.

### A history of the cell ontology

In order to compare experimental results and other information about cell types, a standard reference nomenclature that includes consistent cell type names and definitions is required. The Cell Ontology (CL) is a biomedical ontology that has been developed to provide this standard reference nomenclature for in vivo cell types, including those observed in specific developmental stages in the major model organisms [1]. The semantic hierarchy of CL is mainly constructed using two core relations – *is_a* and *develops_from* – with *is_a* used to relate specific cell subtypes to a more general parent cell type, and *develops_from* used to represent developmental cell lineage relationships.

CL is a candidate for membership in the Open Biomedical Ontology Foundry (OBO Foundry) [2] of reference ontologies. The OBO Foundry is a collective of ontology developers and stakeholders that are committed to collaboration and adherence to shared principles and best practices in ontology development. The mission of the OBO Foundry is to support the development of a family of interoperable biomedical and biological ontologies that are both logically well-formulated and scientifically accurate. To achieve this, OBO Foundry participants adhere to and contribute to the development of an evolving set of principles, including open use, collaborative development, non-overlapping and strictly-focused content, and common syntax and relations.

Masci et al. proposed a major revision to the CL using dendritic cells as the driving biological use case [3]. This revision grew out of a U.S. National Institute of Allergy and Infectious Disease (NIAID)-sponsored “Workshop on Immune Cell Representation in the Cell Ontology,” held in 2008, where domain experts and biomedical ontologists worked together on two goals: (1) revising and developing terms for T lymphocytes, B lymphocytes, natural killer cells, monocytes, macrophages, and dendritic cells, and (2) establishing a new paradigm for a comprehensive revision of the entire CL. The original CL contained a multiple inheritance structure with cell types delineated by a number of different cellular qualities, e.g. “cell by function”, “cell by histology”, “cell by lineage”, etc. The resulting asserted multiple inheritance structure became unsustainable as newly-identified cell types were being added. It was realized that, at least for cells of the hematopoietic system, cells were often experimentally-defined based on the expression of specific marker proteins on the cell surface (e.g. receptor proteins) or internally (e.g. transcription factors), and that these characteristics could be used as the main *differentia* for the asserted hierarchy using the *has_part* relation from the OBO Relation Ontology to relate cell types to protein terms from the Protein Ontology.

Masci et al. developed an approach in which *is_a* classification comprises a single asserted hierarchy based on expressive descriptions of the cellular location and level of expression of these marker proteins using expanded short-cut relations (e.g. *has_plasma_membrane_part, lacks_plasma_membrane_part,* and *has_high_plasma_membrane_amount*) defined in terms of the *has_part* relation [3]. To capture additional information from the original multiple inheritance hierarchy, they used formally defined, property-specific relations, such as *has_function, has_disposition, realized_in,* and *location_of* to construct logical axioms which could subsequently be used by reasoning to computationally produce a richer inferred hierarchy. The end result is a logically coherent asserted framework for defining cell types based on the expression levels of marker proteins, while still capturing important anatomic, lineage, and functional information that might be important characteristics of specific cell types through inference and reasoning. Diehl et al. applied this approach first to cell types of the hematopoietic system and then later to the full CL [4, 5].

In 2016, Diehl et al. reported on the most recent update to the CL in which the content was extended to include a larger number of cell types (e.g. cells from kidney and skeletal tissue) and strategies for representing experimentally-modified cells in vitro [6]. As of June 2016, the CL contained ~2200 cell type classes, with 575 classes within the hematopoietic cell branch alone.

The CL is used as a reference annotation vocabulary for a number of research projects and database resources, including the ENCODE [7] and FANTOM5 (e.g. [8]) projects, and the ImmPort [9] and SHOGoiN/CELLPEDIA [10] databases. Perhaps more importantly, a software package, flowCL, has recently been developed that allows for the automated mapping of cell populations identified from high-dimensional flow and mass cytometry assays to the structured representation of cell types in the CL [11].

### Challenges of extending the cell ontology to accommodate high content single cell phenotyping assays

The pace at which new cell types are being discovered is on the verge of exploding as a result of developments in two single cell phenotyping technologies – high dimensional cytometry and single cell genomics. On the cytometry side, the recent development of mass cytometry provides measurements of over 40 cellular parameters simultaneously at single cell resolution (e.g. [12]), dramatically increasing our ability to monitor the expression and activation state of marker proteins in a variety of cellular systems. On the genomics side, single cell RNA sequencing is allowing for the quantification of complete transcriptional profiles in thousands of individual cells (e.g. [13]), revealing a complexity of cell phenotypes that was unappreciated only a few years ago. In addition, major new research initiatives, like the Human Cell Atlas (www.humancellatlas.org) supported by the Chan Zuckerberg Initiative, are driving the rapid pace of discovery.

As a result, several major challenges have surfaced that are limiting the ability of the knowledge representation community to keep pace with the output from these emerging technologies. First, in the case of targeted phenotyping technologies that interrogate specific subsets of markers, as with flow and mass cytometry, the lack of standardization of which markers should be used to identify which cell types makes it difficult to directly compare the results from different laboratories using different staining panels. Second, in the case of single cell RNA sequencing technologies that interrogate all detectable transcripts in an unbiased fashion, the difficulty in quantitatively and statistically comparing the resulting transcriptional profiles challenges our ability to recognize if we are observing the same cell type or not. In this paper, we will provide examples of how data being generated by these high content experimental platforms are used to identify novel cell types in both blood and brain, propose strategies for how these data can be used to augment the CL, and discuss approaches that could be used to statistically compare quantitative cell type definitions to determine cell type identity.

## Methods

### Automated cell population identification from high dimensional cytometry analysis

The Human Immunology Project Consortium (www.immuneprofiling.org) was established by the U.S. National Institute of Allergy and Infectious Diseases to study well-characterized human cohorts using a variety of modern analytical tools, including multiplex transcriptional, cytokine, and proteomic assays, multiparameter phenotyping of leukocyte subsets, assessment of leukocyte functional status, and multiple computational methods. Our group has focused on the development of computational methods to analyze flow and mass cytometry data in order to objectively quantify and compare known leukocyte cell types, and to discover novel cell subsets. Once these novel cell types are discovered, our philosophy has been to collaborate with the developers of the CL to augment the CL by inclusion of these novel cell types, and then to annotate our results with standard CL terms.

**Fig. 1:**
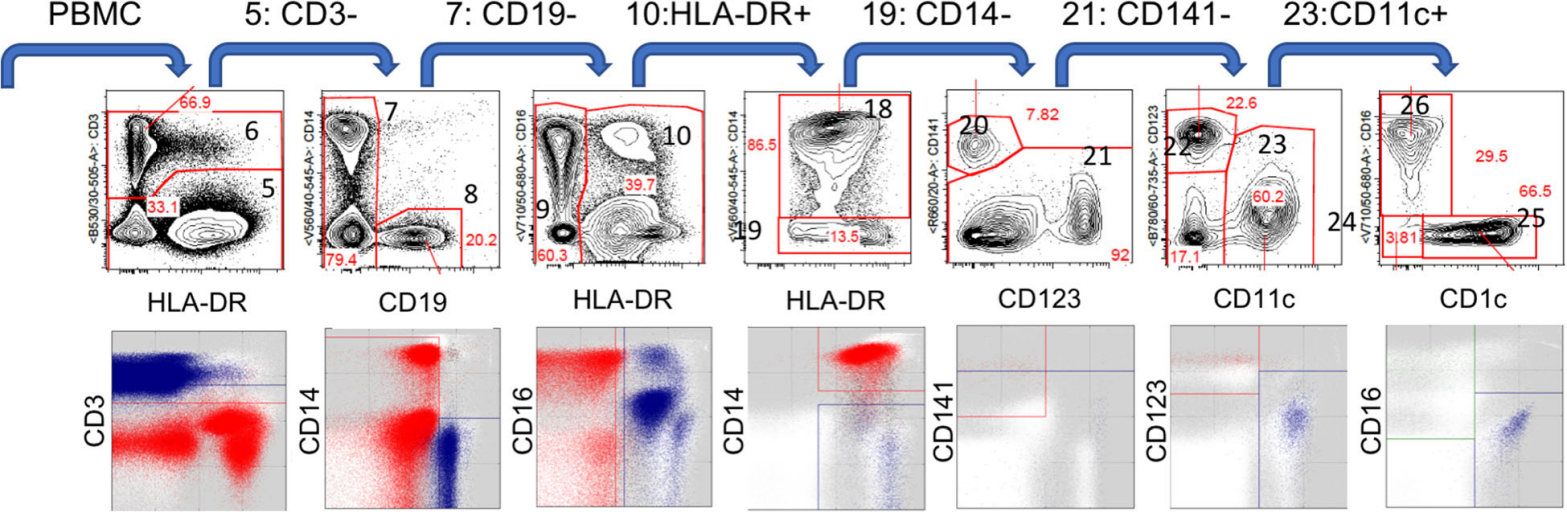
Identification of myeloid cell subtypes using manual gating and directed automated filtering. A gating hierarchy (a series of iterative two-dimensional manual data partitions) has been established by the investigative team in which peripheral blood mononuclear cells (PBMC) are assessed for expression of HLA-DR and CD3, CD3- cells (Population #5) are assessed for expression of CD19 and CD14, CD19- cells (Population #7) are then assessed for expression of HLA-DR and CD16, HLA-DR+ cells (Population #10) are assessed for expression of HLA-DR and CD14, CD14- cells (Population #19) are assessed for expression of CD123 and CD141, CD141- cells (Population #21) are assessed for expression of CD11c and CD123, and CD11c + cells (Population #23) are assessed for expression of CD1c and CD16. Manual gating results are shown in the top panel; directed automated filter results using the DAFi method, a modified version of the FLOCK algorithm [21] are shown in the bottom panel

Figure 1 shows an example of a traditional manual gating hierarchy used to define a subset of myeloid cell subtypes from the peripheral blood of a healthy human donor. In this case, peripheral blood mononuclear cells were stained with a panel of fluorescently-conjugated antibody reagents that recognize a set of cell surface markers that are differentially expressed in a subset of myeloid cell subtypes. A gating hierarchy was established by the investigative team as depicted at the top. From a practical perspective, this gating hierarchy can be thought of as corresponding to the cell type definitions. Applying the cell type names used by the investigative team, the cell type definitions derived from the gating hierarchy would then be:

- Population #18: Monocytes – a PBMC that expresses HLA-DR and CD14, and lacks CD19 and CD3
- Population #19: Dendritic cell (DC) – a PBMC that expresses HLA-DR, and lacks CD14, CD19, and CD3
- Population #20: mDC2 – a dendritic cell that expresses CD141, and lacks CD123
- Population #22: pDC – a dendritic cell that expresses CD123, and lacks CD141 and CD11c
- Population #24: CD1c-CD16- mDC1 – an mDC that expresses CD11c, and lacks CD1c and CD16
- Population #25: CD1c + mDC1 – an mDC that expresses CD11c and CD1c, and lacks CD16
- Population #26: CD16+ mDC – an mDC that expresses CD11c and CD16, and lack CD1c

**Fig. 2:**
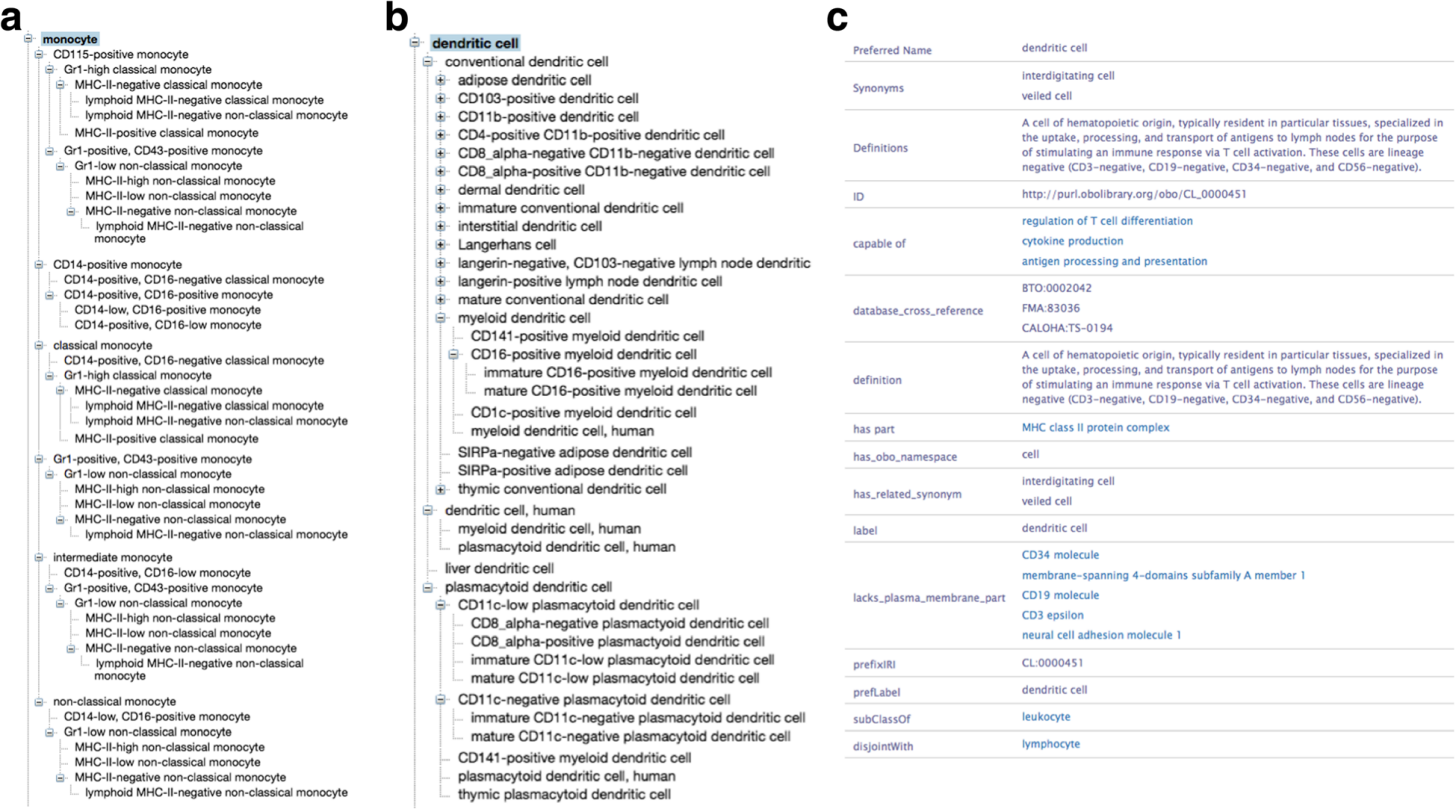
Cell type representations in the Cell Ontology. **a** The expanded *is_a* hierarchy of the monocyte branch. **b** The expanded *is_a* hierarchy of the dendritic cell branch. **c** An example of a cell type term record for dendritic cell. Note the presence of both textual definitions in the “definition” field, and the components of the logical axioms in the “has part”, “lacks_plasma_membrane_part”, and “subClassOf” fields

We attempted to match these experimental cell population definitions to cell types contained in the CL. Figure 2 shows the semantic hierarchy of two major branches in CL for monocytes (A) and dendritic cells (B). Definitions for four of the major relevant cell types from the CL are as follows:

- Monocyte - Morphology: Mononuclear cell, diameter, 14 to 20 μM, N/C ratio 2:1-1:1. Nucleus may appear in variety of shapes: round, kidney, lobulated, or convoluted. Fine azurophilic granules present; markers: CD11b (shared with other myeloid cells), human: CD14, mouse: F4/80-mid, GR1-low; location: Blood, but can be recruited into tissues; role or process: immune & tissue remodeling; lineage: hematopoietic, myeloid. Myeloid mononuclear recirculating leukocyte that can act as a precursor of tissue macrophages, osteoclasts and some populations of tissue dendritic cells.
- CD14-positive monocyte - This cell type is compatible with the HIPC Lyoplate markers for ‘monocyte’. Note that while CD14 is considered a reliable marker for human monocytes, it is only expressed on approximately 85% of mouse monocytes. A monocyte that expresses CD14 and is negative for the lineage markers CD3, CD19, and CD20.
- Dendritic cell - A cell of hematopoietic origin, typically resident in particular tissues, specialized in the uptake, processing, and transport of antigens to lymph nodes for the purpose of stimulating an immune response via T cell activation. These cells are lineage negative (CD3-negative, CD19-negative, CD34-negative, and CD56-negative).
- Myeloid dendritic cell – A dendritic cell of the myeloid lineage. These cells are CD1a-negative, CD1b-positive, CD11a-positive, CD11c-positive, CD13-positive, CD14-negative, CD20-negative, CD21-negative, CD33-positive, CD40-negative, CD50-positive, CD54-positive, CD58-positive, CD68-negative, CD80-negative, CD83-negative, CD85j-positive, CD86-positive, CD89-negative, CD95-positive, CD120a-negative, CD120b-positive, CD123-negative, CD178-negative, CD206-negative, CD207-negative, CD209-negative, and TNF-alpha-negative. Upon TLR stimulation, they are capable of producing high levels of TNF-alpha, IL-6, CXCL8 (IL-8).

The CL monocyte definition includes information about cellular and nuclear morphology, for which we have no information from our flow analysis. The definition of the CD14-positive monocyte is very close to the monocyte cells identified in the flow cytometry experiment in that they are CD14+, CD3- and CD19-. However, since CD20 expression was not evaluated in the panel, we cannot be absolutely certain if the experimental cells represent an exact match to the CL counterpart. Likewise, we cannot determine if the experimental dendritic cell populations match any of the CL dendritic cell populations because CD56 (*a.k.a*. neural cell adhesion molecule 1) expression was not used in the gating hierarchy. Thus, even with semantic assertions of marker protein expression used to formally define cell types (Fig. 2c), exact matching is not possible. Finally, the details of the myeloid dendritic cell definition in CL would be virtually impossible to exactly match since it not only includes a large number of marker expression assertions, but also describes dispositional properties that are difficult to ascertain experimentally.

These findings illustrate a major challenge in the use of automated methods, like flowCL [11], for population matching, which is related to 1) the lack of adoption of standardized staining panels for identification of well-defined hematopoietic cell populations by the research community, even though such staining panels have been proposed [14], and 2) the inconsistent use of experimentally reproducible criteria for cell type definition in CL. A solution to this “partial marker matching” problem is sorely needed.

### Cell population identification from single cell transcriptional profiling

While flow cytometry relies on detection of a pre-selected set of proteins to help define a cell’s “parts list”, transcriptional profiling uses unbiased RNA detection and quantification to characterize the parts list. Recently, the RNA sequencing technology for transcriptional profiling has been optimized for use on single cells, so-called single cell RNA sequencing (scRNAseq). The application of scRNAseq on samples from a variety of different normal and abnormal tissues is revealing a level of cellular complexity that was unanticipated only a few years ago. Thus, we are experiencing an explosion in the number of new cell types being identified using these unbiased high-throughput/high-content experimental technologies.

**Fig. 3:**
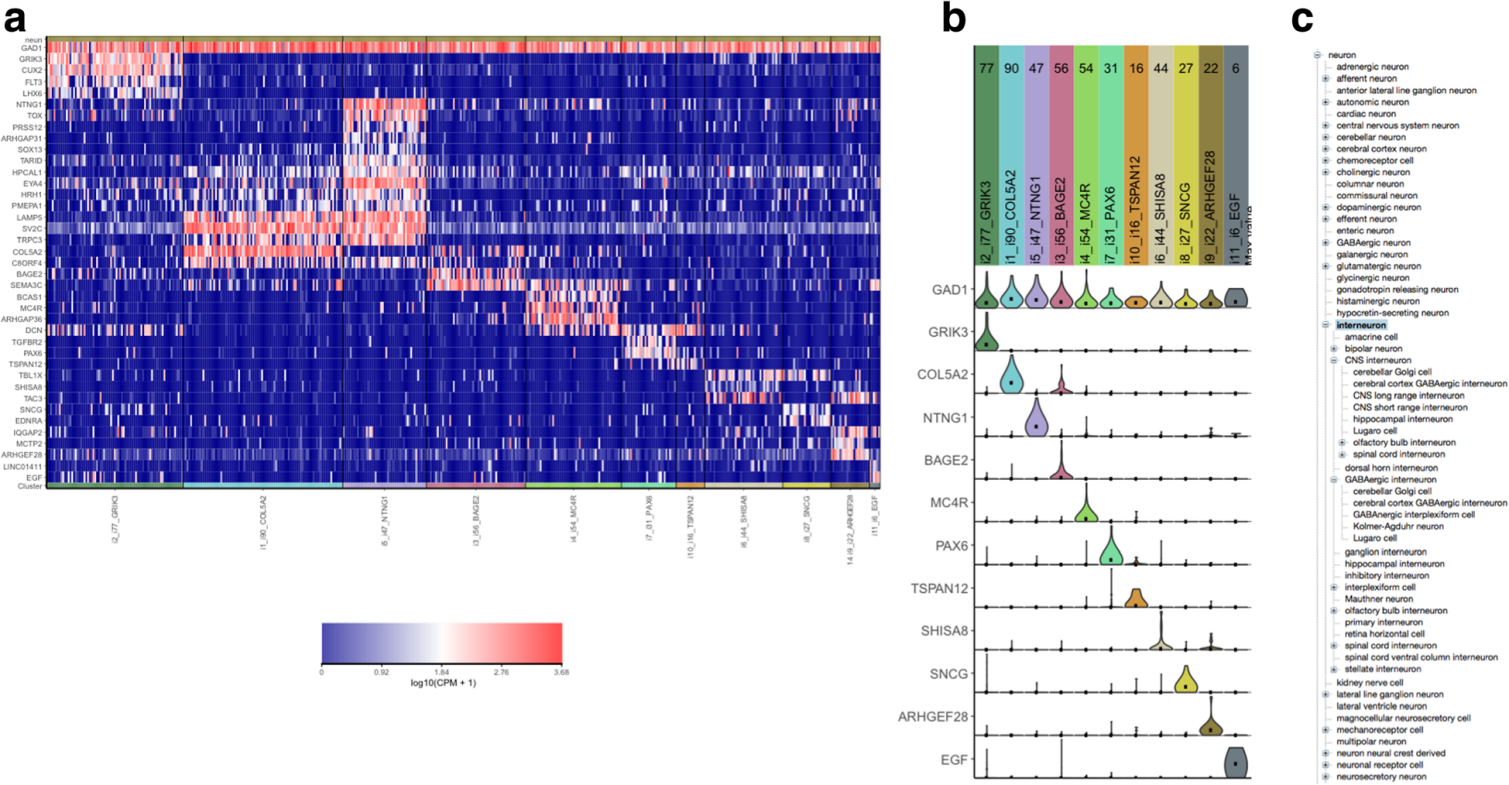
Cell type clustering and marker gene expression from RNA sequencing of single nuclei isolated from layer 1 cortex of post-mortem human brain. **a** Heatmap of CPM expression levels of a subset of genes that show selective expression in the 11 clusters of cells identified by principle component analysis (not show). An example of the statistical methods used to identify cell clusters and marker genes from single cell/single nuclei data can be found in [13]. **b** Violin plots of selected marker genes in each of the 11 cell clusters. **c** The expanded *is_a* hierarchy of the neuron branch of the Cell Ontology, with the interneuron sub-branch highlighted

As an example, our group has recently completed an analysis of the transcriptional profiles of single nuclei from post-mortem human brain using single nucleus RNA sequencing (snRNAseq). Single nuclei from cortical layer 1 of the middle temporal gyrus were sorted into individual wells of a microtiter plate for snRNAseq analysis, and specific cell type clusters identified using iterative principle component analysis (unpublished). A heatmap of gene expression values reveals the differential expression pattern across cells from the 11 different neuronal cell clusters identified (Fig. 3a). Note that cells in all 11 clusters express GAD1 (top row), a well-known marker of inhibitory interneurons. Violin plots of selected marker genes for each cell cluster demonstrate their selective expression patterns (Fig. 3b). For example, GRIK3 is selectively expressed in the i2 cluster.

In order to determine if the distinct cell types reflected in these snRNAseq-derived clusters have been previously reported, we examine the neuronal branch of the CL (Fig. 3c) and found that the cerebral cortex GABAergic interneuron is probably the closest match based on the following relevant definitions:

- cerebral cortex GABAergic interneuron - a GABAergic interneuron that is part_of a cerebral cortex.
- GABAergic interneuron – An interneuron that uses GABA as a vesicular neurotransmitter.
- interneuron – Most generally any neuron which is not motor or sensory. Interneurons may also refer to neurons whose axons remain within a particular brain region as contrasted with projection neurons which have axons projecting to other brain regions.
- neuron - The basic cellular unit of nervous tissue. Each neuron consists of a body, an axon, and dendrites. Their purpose is to receive, conduct, and transmit impulses in the nervous system.

Given these definitions, it appears that each of the cell types defined by these single nuclei expression clusters represents a novel cell type that should be positioned under the cerebral cortex GABAergic interneuron parent class in the CL.

### Cell types versus cell states

A fundamental issue has also emerged in considering how to distinguish between discrete *cell types* and more fluid *cell states*. It is clear that, in addition to the programmed process of cellular differentiation, cells are constantly responding and adapting to changes in their environment by subtly changing their phenotypic states. In the case of the hematopoietic system, cells are frequently responding to their environment to activate specific effector functions in order to re-establish normal homeostasis. The question is, does the phenotypic cellular change that characterizes this response represent a new *cell type* or not?

## Results and Discussion

These examples of cell population identification using two different single cell phenotyping technologies have illustrated a number of challenges emerging with these high-throughput/high-content assay platforms, including:

- matching cell populations identified using assay platforms focused on molecular expression with cell types represented in the reference CL ontology that have been defined using other non-molecular characteristics;
- matching cell populations identified using overlapping but non-identical marker panels;
- adding new cell populations being rapidly identified with these high-throughput assay platforms to a reference ontology in a timely fashion;
- determining what kind of validation would be required to add a novel cell type to a reference ontology;
- determining if a standard naming and definition convention could be developed and adopted;
- distinguishing between truly discrete cell types and responsive cell states.

We conclude by presenting a series of proposals for consideration to address these challenges.

**Fig. 4:**
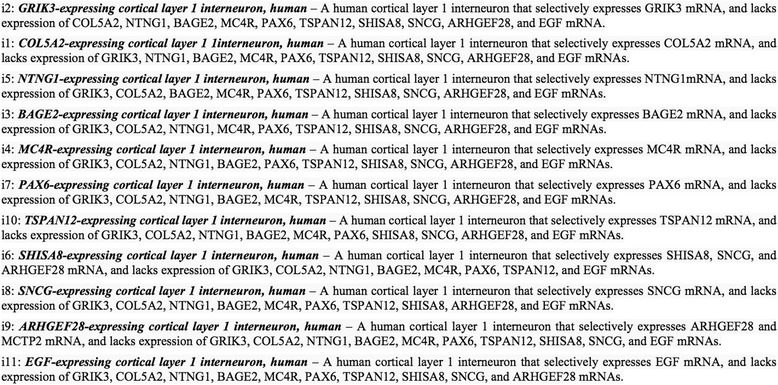
Proposed cell type names and definitions for cell types identified from the snRNAseq experiment shown in Fig. 3

1. *Establish a new working group* – We propose the establishment of a new working group composed of CL developers and representatives of the Human Cell Atlas group and other stakeholder communities to develop strategies for naming, defining, and positioning new cell types identified through high throughput experiments in the CL.
2. *Molecular phenotype-based definitions* – The community should continue to focus cell type definitions in the CL on precisely describing the phenotype of the cells, molecular and otherwise, using a series of necessary and sufficient conditions expressed as logical axioms.
3. *Evidence requirements for inclusion in CL* - The CL developers should consider the development of policies regarding the veracity of support required for the addition of a new cell type into the CL reference ontology, including whether a single report is sufficient, or whether some form of independent validation should be required.
4. *Provisional CL -* If independent validation is required, the CL developers should consider the establishment of a “CL provisional ontology” that could be used to hold provisional cell type assignments while they are being fully validated using the criteria defined in addressing Proposal #3.
5. *Inclusion of experimental context* - As cell type discovery experiments become more and more sophisticated, it will be essential to capture information about the experimental context in which the cells were initially identified. Thus, cell type definitions should also include “context annotations” in the form of standardized experiment metadata along the lines of the MIBBI [15] and OBI [16] minimum information and vocabulary standards, respectively.
6. *Incomplete overlapping of assessed phenotypes* - In the case of similar cell types identified by overlapping staining panels in flow and mass cytometry experiments, identify the most common parent class and define the child classes based on the specific markers that were actually evaluated in the experiment. For example – the “CD14+, HLA-DR+, CD19-, CD3-, peripheral blood mononuclear cell monocyte” identified in the above experiment would be positioned as a child of a new “CD14+, CD19-, CD3- monocyte” parent, and as a sibling to the current “CD14-positive monocyte” defined in the CL, whose name and definition would need to be changed to “CD14+, CD20+, CD19-, CD3- monocyte”, since we don’t know about the expression of CD20 in the former or the expression of HLA-DR in the latter.
7. *Cell types from single cell transcriptomics* - Given the rapid expansion in the application of single cell transcriptional profiling for novel cell type identification, it will be critical to develop conventions for cell type naming and definition using data from transcriptional profiling experiments. For example, the 11 new cell types identified in Fig. 3 could be named by combining marker genes selectively expressed by the cells with the parent cell class and the context (tissue specimen and species source) in which the cell types were identified, as shown in Fig. 4.
8. *Selection of useful marker genes* - When cell types are identified using gene expression-based clustering approaches, it is useful to select a set of marker genes that are informative for cell type identification in a given dataset. Several different approaches have been used to select genes for cell type clustering, including simple approaches like genes with the highest variance across a dataset, or more sophisticated methods like the genes contributing to the top principle components in a PCA analysis, or genes that serve as the most useful features in a machine learning-based classification model. For example, in a recent method used to test cell lines for pluripotency [17], Muller et al. proposed the use of non-negative matrix factorization to select out multi-gene features for characterizing the stem cell phenotype. These marker genes can then be used to specify the cell type definition.
9. *Marker gene selectivity* - The naming and definition convention presented in Fig. 4 derives from the computational analysis of experimental data to identify marker genes that show “specific” expression in each of the cell type clusters. In this case, “specific” is a relative, rather than absolute, term indicating that the marker gene is expressed at a significantly different level in one cell type than in the other cell types assessed in the experiment. In addition, we will often have incomplete knowledge about the expression of this marker gene in all other cell types in the complete organism. Thus, we have included in the definition the “selectively” qualifier to indicate relative specificity, and the starting source material (i.e. cortical layer 1) to indicate the subsystem evaluated in the experiment.
10. *Necessary and sufficient conditions* – Ideally, each cell type would be defined by the necessary and sufficient conditions that uniquely distinguish the cell type from all other cell types in the complete organism. In the proposed definitions described in Fig. 4, we selected a single positive marker gene for each of the 11 cell type clusters identified, and include a statement about the relative absence or presence of all marker genes in each cell type definition. However, it is not clear if it is necessary to explicitly include the absence of expression of all ten negative marker genes; it may be sufficient, at least for some cell types, to state the selective expression of one positive marker gene and the absence of expression of one negative marker gene to adequately define the cell type in question. Some further exploration on how best to determine the necessary and sufficient conditions of marker gene expression for cell type definitions is required.
11. *Use of negative assertions through “lacks expression of*” – For many cell types, providing necessary and sufficient conditions requires asserting that the cell type does not express a molecule. Consistent with the approach taken by the CL ontology, we have used “lacks expression of” in our natural language definitions (Fig. 4). In formal assertions, the CL uses the relation *lacks_part*. The “lacks” relations are considered “shortcut” relations that must be translated to formal expressions that can be interpreted appropriately by logical reasoners [18, 19]. Thus, the CL translates “X *lacks_part* Y” to the OWL expression “X subClassOf has_part exactly 0 Y” [5].
12. *Cell type matching* - The informatics community will also need to develop statistically-rigorous methods for the comparison of datasets to match equivalent cell types identified in independent experiments. For example, our group has described the implementation and use of the Friedman-Rafsky statistical test in the FlowMap-FR tool for cross-sample cell population matching from flow cytometry data [20]. This type of approach could be explored for comparing multivariate expression profiles to determine how similar they are to each other. An alternative strategy has been proposed by Muller et al. [17] in which the results from two complementary logistic regression classifiers are combined for sample classification against a reference database of relevant cell type expression data. As the field moves forward, these types of statistically-rigorous approaches for expression data-based comparative classification will be essential.
13. *Cell types* versus *cell states* - Our intuition is that there is a distinction between discrete cell types that might be generated as a result of programmed differentiation and more subtle changes in cell states experienced by a given cell type in response to changes in its environment. The challenge is to come up with a coherent and consistent approach for making this distinction. Although new cell types and new cell states reflect phenotypic changes that occur through temporal processes, we propose that the distinction relates to the stability and reversibility of the new cellular phenotype. Thus, the generation of a distinct cell type through the process of programmed differentiation is not only stable but also irreversible under normal circumstances. In contrast, a change in cell state is only stable in a certain environment and is reversible with a change in that environment. As an example, the transition from a naïve to memory T cell is an example of a change in cell type through differentiation, in that it reflects a stable and irreversible change (once you’ve experienced antigen, there’s no going back). In contrast, activating a memory T cell in response to antigen exposure would be considered a change in state, in that once the stimulus has been eliminated, the memory T cell would return back to its initial state. Thus, an activated memory T cell would be considered a change in state of a memory T cell rather than a new cell type.

## Conclusions

The advent of high-throughput/high-content single cell technologies is leading to an explosion in the number of distinct cell types being identified. This development is resulting in several significant challenges in efforts to reproducibly describe reference cell types for comparative analysis. Over the next couple of years, it will be critical for the bioinformatics community to develop and adopt data standard conventions that will be compatible with these new technologies and support the data representation needs of the research community. The proposals enumerated here should serve as a useful starting point for this work.

## Abbreviations

CL: Cell Ontology
MIBBI: Minimum Information for Biological and Biomedical Investigations
OBI: Ontology for Biomedical Investigations
OBO: Open Biomedical Ontology
scRNAseq: single cell RNA sequencing
snRNAseq: single nucleus RNA sequencing
